# The Institutional Worker

**Published:** 1919-12-27

**Authors:** 


### INSTITUTIONAL WORKER

**Being a Special Supplement to "The Hospital"**

### OUR BUREAU OF INFORMATION.

### Rules for Correspondents.

1. Every letter must be accompanied by the coupon to be cut from the back cover (inside page) of The Hospital, current issue, and must contain the name and address of the correspondent with pseudonym for publication if desired. Replies by post cannot be given save under exceptional circumstances at the Editor's discretion.
2. Letters from Approved Homes in reply to special needs published in the Bureau should state terms and full particulars, and be sent prepaid under cover to the Editor of the Bureau with name written across coupon for identification.
3. Proprietors of Homes which have not yet been entered on the List of Approved Homes, but have spare accommodation likely to suit special needs, are invited to write for an application form for registration. The fee for registration, which includes two announcements of the Home in the Bureau and other privileges, is 10s.
4. All communications to be addressed to the Editor of The Hospital, 28 Southampton Street, Strand, London, W.C. 2, and marked "Bureau of Information."

### INSTITUTION FACTS AND FIGURES.

### The Question Box.

### REGULATING A PATIENT'S VISITORS.

The question was:

**Elaborate a plan whereby the number of patients' visitors may be regulated on visiting days, so that none may be standing about the corridors and all may see the patient in turn. Each patient to be allowed six visitors, but not more than two at a time.**

**H. L. replies:**

If the duration of visiting be one hour, since the maximum number of visitors allowed is six, and not more than two are permitted to see a patient at a time, the length of stay of visitors will be twenty minutes, half-hour, or one hour, according as the number of such is six or five, four or three, or two or one.

Two cards should be issued by the ward sister to a patient, who will determine the number of visitors (not exceeding six) he or she desires to see, and the times at which they shall attend during the hour.

On visiting day there must be but one entrance to the building possible, say the lodge, the receiving room or out-patient hall, according to convenience, all other means of ingress being closed. The visitors who are to be the first to see patients must produce their cards to a porter, or porters, on duty at the entrance, and proceed to the wards. At the expiration of twenty minutes a bell should be rung, indicating that those of the patients who are having six or five visitors shall request those in the wards to retire in order that the second pair be admitted. The nursing staff on duty in the several wards will, of course, co-operate in this.

The visitors leaving the wards will return to the entrance and hand their cards to the other relatives or friends who are due to see the patient, and the second batch will now proceed to the wards, a process which will be repeated at the half-hour, and finally after the lapse of forty minutes. If a patient has but one or two visitors, such will stay in the ward during the whole of the hour.

There will not be any congestion along the corridors, because the second and third batches of visitors will only be admitted by means of the cards which the departing persons hand to them as they leave the place of both entrance and exit. Moreover, the rule as to the number of visitors can be the better enforced.

An alternative method is to have different coloured cards to denote the times for visitation, but such a system has the following defects:

1. The likelihood of more than the maximum number of visitors to a patient being admitted, the only check being at the wards, which would involve no little trouble.
2. If the place of entrance and exit were one, congestion and delay would ensue owing to the incoming and departing persons passing simultaneously along the corridors.
3. Even if congestion and delay were avoided by having an exit separate from the entrance, an extra porter, or porters, would be necessary for duty thereat.
4. Additional printing expenditure would be involved.

### THE STAFF REQUIRED FOR A FEVER HOSPITAL.

It is often difficult for the matron of a small hospital, whether general or special, who is anxious to administer the institution on the most up-to-date lines to determine the basis by which her staff may be calculated. Several inquiries have reached us lately as to the staff required to work a country fever hospital, and we think that the following information, drawn up according to the regulations of the Metropolitan Asylums Board, may be of assistance and interest.

In accord with the most modern developments, the Board assume that the staff works forty-eight hours a week only. Overtime is paid for at the rate of time and a quarter for the first two hours, and time and a half thereafter. For a hospital with fifty beds (in six wards), allotting two wards each of fifteen to the cases with scarlet fever, two wards each of seven beds to diphtheria, and two wards each of three beds to enteric, the staff should number twenty-six nurses (exclusive of matron) and eight wardmaids, a total in all of thirty-five. Each scarlet-fever ward should have three nurses, with three between the two for relief, making a total of nine, with one wardmaid for each ward and one between the two, making a total of three. For the diphtheria wards the same number of nurses and wardmaids should be provided as for the scarlet-fever wards. The enteric wards should have three nurses each and two between the two for relief, making a total of eight, and one wardmaid for the two wards, with one for relief, making a total of two. In staffing the wards much of course depends on the position of each and the relation of each to the rest.

Then comes the question of salaries. The Metropolitan Asylums Board believes in adequately remunerating its employees, and the rate of salaries suggested as follows:—Sisters, £44, rising to £50 by annual increments of £3; staff nurses, £33, rising by £3 to £39; probationers, £22, rising by £2 to £24; wardmaids, £22. In addition every member of the staff is paid a war bonus of £18, bringing the salaries up to the following rates:—Sisters, £62; staff nurses, £51; probationers, £40; wardmaids, £40, with board, lodging, washing, and uniform in each case.

### Question for December.

### A COTTAGE HOSPITAL LAUNDRY.

**For a cottage hospital containing fifty beds, and a staff of twelve including matron, nurses, and maids, give details of size of laundry necessary, also full particulars of the equipment, machinery, and staff required, and some idea as to the cost of working.**

**A minim um payment of 10s. 6d. will be made for each published answer.)**

RULES.

The following rules must be observed:—

1. Contributions must be written on one side of the paper. Conciseness and terseness are desirable features. The MS. must bear the name and address of the sender and be accompanied by coupon to be cut from the back cover (inside page) of the current issue of The Hospital. A pseudonym must be chosen if the name is not to be published.

2. Contributions must be addressed to the Editor of The Hospital Institutional Worker Supplement, 28 & 29 Southampton Street, Strand, London, W.C. 2, should reach him before the end of the current month, and be marked in the left-hand corner "Facts and Figures."

### QUESTIONS INVITED.

In connection with our Question Box, we offer every month five shillings each for the best questions which are sent in for consideration. The questions may be on any institutional subject and concern any department, from the secretary's to the porter's, or the matron's to the domestic staff; the one essential is that they must be practical and deal with points that have a definite relation to hospital or institutional administration, upkeep, finance, or management. Points relating to artificers' work, housekeeping, the laundry, out-patients, and other practical matters will be welcome. It is hoped that thus, when difficult or doubtful points arise in the course of their work, institutional workers will be encouraged to put them in the form of a question and send them to the Editor, The Hospital Bureau of Information, 28 & 29 Southampton Street, Strand, London, W.C. 2, marked "Question Box."

The best questions will be published in due course and our readers will be given the opportunity of answering them. Thus all institutional workers may be able to co-operate with the view of helping the work and smoothing the difficulties of each other. In short, we want the Question Box of the *Institutional Worker* to be freely used by every worker habitually.

### ENQUIRIES AND ANSWERS.

*SICK AND IN NEED.*

### Mental Case.

Private patients are received at the City of London Mental Hospital, Dartford, Kent. The fees range from 28s. per week and upwards. Application should be made to the Medical Superintendent. The Coton Hill Mental Hospital, near Stafford, also provides accommodation for the treatment of the mentally afflicted of the middle classes. —A. C.

*EMPLOYMENT AND TRAINING.*

### Nursing in Egypt.

We suggest that you write to the following institutions and inquire if there are any vacancies on their staff: Anglo-American Nursing Home, Cairo; Victoria Nursing Home, Cairo; Kasr-el-ainy Hospital, Cairo; and the Greek Hospital, Alexandria.—Carew.

### Prospects of Nursing Home.

Circumstances in some respects favour prospects of successfully commencing and maintaining a nursing home. During the past year or so most well-managed homes in London have found it exceedingly difficult to meet all the demands for accommodation. Provision for maternity cases is even more difficult to obtain, owing probably to the increased demand through the lack of convenient housing. You do not state whether you hold a certificate of general training. The absence of this certificate would preclude you from maintaining a medical and surgical home unless you have a partner who is professionally competent. Your qualifications and the needs of the times suggest a maternity home where patients could be received for seven or eight guineas a week.

Unless you have most of the necessary furniture, it would be quite impossible for you to equip a home of six to ten beds for £400. A capital of from £600 to £1,000, provided you can look to your relatives for personal maintenance until the home pays, might make it just possible. We advise you carefully to weigh all the facts. Your first thought must be to guard against loss, which could be very readily incurred in an undertaking of this character. You must not forget the dead expenses, which must be paid whether your house is full or empty, such as rent, rates, taxes, domestic services, fuel and lighting, and furniture repairs and replacements.—H. M. W.

### Position as Nurse Companion.

These columns are reserved for giving information to those who seek it; we are unable to undertake the duties of an Employment Bureau. The only course feasible or possible is to advertise your need, or reply to the advertisements which appear in the nursing journals and daily papers.—"Nurse N."

### Massage and Electricity Course.

The length of time for training in massage has been increased to twelve months instead of six, beginning in 1920, by the Incorporated Society of Trained Masseuses. It is certainly an advantage to take the course at a hospital, although it is possible to obtain the necessary instruction from a private teacher, the student attending a hospital for the necessary practical experience. The usual fee for instruction is £15 15s. The electricity course extends over three months, the fee for which is £5 5s. As you suggest, the number of trained masseuses far exceeds the demand, and even if you have a large circle of medical friends it takes at least a year to build up a practice. Write to the Secretary of the Incorporated Society of Trained Masseuses, 157 Great Portland Street, London, W., who will furnish you with names of training schools and private teachers.—Masseuse.

*MISCELLANEOUS.*

### Temporary Building.

We cannot say with any certainty that the London County Council would give you licence to put up a structure other than a brick building to accommodate your cases for physical treatment, but it is open to you to make application to the Council through their Superintending Architect, setting forth your difficulties from the financial standpoint. Your application should be accompanied by a preliminary block plan and elevation of the structure you propose to erect, together with a fee of 5s. Details of the materials to be used should be set forth, and if these materials are more or less fireproof, such as Poilite slabs on wood frame, with galvanised-iron roof, the authorities might under certain circumstances grant a licence.—Secretary.

### Artificial Limbs.

The Essential Artificial Limb Co., of 24 South Molton Street, W. 1, are makers of artificial limbs possessing all-round good qualities. In these limbs utility, comfort, and simplicity are combined, and we suggest that you personally inspect the work of this firm. A stump socket which is capable of adjustment to changes in the size of the stump, so essential to comfort, is a special feature of these limbs, and perfect control over jointed limbs is secured without complicated or delicate mechanism. The Hobbs' mechanical arm is a speciality of this firm, and for the worker has already proved to be a great boon.—W. H.

### Nursing Service.

Apart from home visiting and special welfare work arranged by municipal authorities, we know of no general district nursing for the poor which is paid for out of the rates. We suggest that you write to the Ministry of Health.—Nic.

